# Identification of HN252 as a potent inhibitor of protein phosphatase PPM1B

**DOI:** 10.1111/jcmm.15975

**Published:** 2020-10-13

**Authors:** Zhiyuan Lu, Peng Xiao, Yuan Zhou, Zhenyu Li, Xiao Yu, Jinpeng Sun, Yuemao Shen, Baobing Zhao

**Affiliations:** ^1^ Key Laboratory of Chemical Biology (Ministry of Education) School of Pharmaceutical Sciences Cheeloo College of Medicine Shandong University Jinan China; ^2^ Key Laboratory Experimental Teratology of the Ministry of Education and Department of Biochemistry and Molecular Biology School of Medicine Cheeloo College of Medicine Shandong University Jinan China; ^3^ Department of Pharmacy Shandong Provincial Hospital Cheeloo College of Medicine Shandong University Jinan China; ^4^ Department of Pharmacology School of Pharmaceutical Sciences Cheeloo College of Medicine Shandong University Jinan China

**Keywords:** inhibitor, PPM1B, protein phosphatase, p‐terphenyl, substrate

## Abstract

Protein phosphatase 1B (PPM1B), a member of metal‐dependent protein serine/threonine phosphatase family, is involved in the regulation of several signalling pathways. However, our understanding of its substrate interaction and physiological functions is still largely limited. There is no reported PPM1B inhibitor to date. In this study, we identified HN252, a p‐terphenyl derivative, as a potent PPM1B inhibitor (*K_i_* = 0.52 ± 0.06 µM). HN252 binding to PPM1B displayed remarkable and specific inhibition of PPM1B in both in vitro and ex vivo. With the aid of this small molecular inhibitor, we identified 30 proteins’ serine/threonine phosphorylation as potential substrates of PPM1B, 5 of which were demonstrated by immunoprecipitation, including one known (CDK2) and 4 novel ones (AKT1, HSP90B, β‐catenin and BRCA1). Furthermore, GO and KEGG analysis of dramatically phosphorylated proteins by PPM1B inhibition indicated that PPM1B plays roles in the regulation of multiple cellular processes and signalling pathways, such as gene transcription, inflammatory regulation, ageing and tumorigenesis. Our work provides novel insights into further investigation of molecular mechanisms of PPM1B.

## 1 INTRODUCTION

Protein phosphorylation plays a crucial role in regulating numerous cellular processes.^1^, ^2^, ^3^ Protein phosphatases catalyse the removal of phosphate groups from proteins, reversing the action of protein kinases. The fully sequenced human genome is thought to contain 518 putative protein kinases and only around 189 putative phosphatases.^4^, ^5^ This imbalance in the number indicated that protein phosphatases are less specific and less tightly regulation compared to protein kinases. Indeed, kinases have been well studied and established targets for drug discovery. In contrast, the knowledge of phosphatase function, regulation and substrate interaction is still largely limited.^6^

According to substrate specificity and mechanism, protein phosphatases are simply classified into two different families: protein serine/threonine phosphatases (PSPs) and protein tyrosine phosphatases (PTPs).^7^ PSPs mainly include phosphoprotein phosphatases (PPPs) and the metal‐dependent protein phosphatases (PPMs), which catalyse the majority of phosphoserine (pSer) and phospho‐threonine (pThr) dephosphorylation in eukaryotic cells. Although the inhibitors of PTPs are widely studied, modulators of most PSPs are rarely documented because of the poor specificity and regulation, which keeps it away from phosphatase‐directed therapeutics.^6^, ^8^ Nevertheless, these identified molecules serve as powerful tools to characterize the physiological roles of the corresponding enzymes, making them therapeutically attractive lead compounds. Recently, several protein phosphatases have been validated as therapeutic targets for various diseases, such as PPM members PP2Cα in liver fibrosis, WIP1 in cancer and PHLPP2 in diabetes and heart disease.^8^

Protein phosphatase 1B (PPM1B) is a member of PPM superfamily that are Mg^2+^/Mn^2+^‐dependent monomeric phosphatases.^9^ It has been demonstrated to be involved in several signalling pathways depending on the cellular context, such as MAPK signalling,^10^ AMPK signalling,^11^ TNF signalling,^12^, ^13^ gene transcription^14^ and cell cycle.^15^ However, its physiological functions and the biological relevance in the human diseases are still unclear. To date, there is no reported PPM1B inhibitor. In this study, we demonstrated that HN252, a p‐terphenyl derivative, is a novel inhibitor of PPM1B and also identified potential substrates and relative signalling pathways for PPM1B.

## 2 MATERIALS AND METHODS

### 2.1 Antibodies and reagents

HN252 was obtained according to the similar method described previously.^16^ Detailed information on the antibodies and reagents used in this study is shown in the Supplementary Information (Table S8).

### 2.2 Cell culture

HL7702, COS7, PC‐3 and HCT116 cells were purchased from the Cell Bank of the Shanghai Institute for Biological Sciences, Chinese Academy of Science. HCT116, COS7 and PC3 cells were cultured in DMEM (Gibco); HL7702 cells were cultured in RPMI 1640 medium (Gibco). All media were supplemented with 10% foetal bovine serum (Biological Industries), 100 IU penicillin and 100 mg/mL streptomycin at 37°C in a humidified incubator with 5% CO_2_.

### 2.3 Protein purification and kinetic assays of protein phosphatases

*Escherichia coli* BL21 strain was transformed with pET‐28a bacterial expression vector harbouring PPM1B and its mutants.^17^ The fusion proteins were induced to be expressed with 0.5 mM isopropyl‐β‐d‐thiogalactoside (IPTG) and purified from bacterial lysate by Ni‐NTA chromatographic matrices (Qiagen) and AKTA FPLC (GE Healthcare Life Sciences) using a Superdex Increase 200 column.

The kinetic assays of PPM1B were carried out at 37°C in 0.1 M acetic acid buffer (50 mM Tris, 40 mM Mn^2+^, pH 7.0). The p‐nitrophenyl phosphate (pNPP, Sigma) was used as the substrate which could be conveniently measured at 405 nm. Reactions were started by addition of pNPP to a reaction mixture containing PPM1B (0.4 μM PPM1B, final concentration) and various concentrations of HN252 and stopped by addition of 0.5 M EDTA (pH 10.0) after an appropriate time. The *K_m_* value of PPM1B towards pNPP hydrolysis (3.6 mM for pNPP) was used to determine the IC_50_. The IC_50_ values, the inhibition constant *K_i_* and inhibition pattern were evaluated as previously described.^18^ The kinetic assays of HN252 towards to other protein phosphatases were carried out as previously described.^19^

### 2.4 Fluorescence quenching assay

The interaction between PPM1B recombinant proteins and compound HN252 was determined by fluorescence titration as previously described.^20^ PPM1B proteins were dialysed against PBS and incubated with different concentrations of HN252. The fluorescent quenching was conducted at 25°C, and fluorescence spectra were recorded by Cytation 5 Multi‐Mode Plate Reader (BioTek) using excitation at 280 nm and emission at 332 nm. The dissociation constant (*K_d_*) of HN252 to PPM1B was calculated as previously described.^20^

### 2.5 Molecular modelling

Molecular docking study was performed to investigate the binding mode between the HN252 and the human PPM1B using AutoDock Vina 1.1.2.^21^ The three‐dimensional (3D) structure of the PPM1B (PDB ID: 2P8E) was downloaded from the RCSB Protein Data Bank (www.rcsb.org). The 3D structure of the HN252 was drawn by ChemBioDraw Ultra 14.0 and ChemBio3D Ultra 14.0 software. The AutoDockTools 1.5.6 package was employed to generate the docking input files.^22^ The binding site of the PPM1B was identified as center_x: 12.763; center_y: 19.031; and center_z: 37.015 with dimensions size_x: 15; size_y: 15; and size_z: 15. In order to increase the docking accuracy, the value of exhaustiveness was set to 20. For Vina docking, the default parameters were used if it was not mentioned. Then, an MD study was performed to revise the docking result.

The Amber 12 and AmberTools 13 programs were used for MD simulations of the selected docked pose as previously described.^23^ HN252 was first prepared by ACPYPE for generating automatic topologies and parameters in different formats for different molecular mechanics programmes, including calculation of partial charges. Then, the force field ‘leaprc. gaff’ (generalized amber force field) was used to prepare the ligand, whereas ‘leaprc. ff12SB’ was used for the receptor. The system was placed in a rectangular box (with a 10.0 Å boundary) of TIP3P water using the ‘SolvateOct’ command with the minimum distance between any solute atoms. Equilibration of the solvated complex was done by carrying out a short minimization (2000 steps of each steepest descent and conjugate gradient method), 1000 ps of heating and 500 ps of density equilibration with weak restraints using the GPU (NVIDIA^®^ Tesla K20c)‐accelerated PMEMD (Particle Mesh Ewald Molecular Dynamics) module. At last, 100 ns of MD simulations was carried out. The binding free energies (ΔG_bind_ in kcal/mol) were calculated using the Molecular Mechanics/Generalized Born Surface Area (MM/GBSA) method, implemented in AmberTools 13. For each complex, the binding free energy of MM/GBSA was estimated as follows: ΔG_bind_ = G_complex_‒G_protein_‒G_ligand_, where ΔG_bind_ is the binding free energy, and G_complex_, G_protein_ and G_ligand_ are the free energies of complex, protein and ligand, respectively.

### 2.6 RNA interference and lentivirus infection

Lentiviral shRNAs were constructed into pLKO.1‐puro vector following the manufacturer's instructions (Thermo Scientific). The indicated shRNA targeting sequences are as follows: shScramble: 5′‐CGCTGAGTACTTCGAAATGTC‐3′; shPPM1B: 5′‐GACTGAATCCACATAGAGAAA‐3′; and shPPM1A: 5′‐GCACCCAAAGTATCGCCAGAA‐3′. For lentivirus production, 293T cells were transfected with lentiviral vectors carrying shRNAs and lentivirus‐packing plasmids (psPAX2/pMD2G) using the LipoFiter™ reagent according to the manufacturer's protocols. Virus‐containing medium was collected after 48h and added to cells with fresh medium containing 10 μg/mL polybrene. The virus‐containing medium was then replaced with fresh medium after 12‐h culture.

### 2.7 Western blot analyses

Cells were lysed in lysis buffer (50 mM Tris‐HCl, 150 mM NaCl, 0.1% SDS, 1% NP‐40, 0.5% deoxycholate, pH 7.4), supplemented with protease inhibitor (Roche) and phosphatase inhibitor cocktails (Roche). Protein samples were separated by SDS‐PAGE and wet‐transferred to PVDF membranes (Millipore). The membrane was blocked for 1 h in 5% BSA in TBST at room temperature and probed with the appropriate primary antibodies and with an HRP‐conjugated secondary antibody.

### 2.8 Phosphorylation profiling

Phosphorylation profiling was performed by using the Phospho Explorer Antibody Microarray from Full Moon BioSystems, Inc (Sunnyvale, CA), according to the protocol which was previously described.^24^ In brief, cell lysates obtained from HL7702 cells treated by HN252 or DMSO for different hours or transfected with lentiviral vectors carrying shRNAs for 36 h were biotinylated with the Antibody Array Assay Kit (Full Moon BioSystems, Inc), and the data were analysed with the GenePixTM Pro 4.0 image analysis software.

### 2.9 Immunoprecipitation and mass spectrometry analysis

The lysates of HL7702 cells overexpressed pCDH‐HA‐PPM1B or pCDH blank vector were subjected to immunoprecipitation experiments using Anti‐HA Magnetic Beads according to the manufacturer's protocols. For mass spectrometry analysis, the eluted proteins were digested with trypsin and analysed by a Q Exactive HF‐X (Thermo Scientific) mass spectrometer. Peptides were identified by using MASCOT search engine (Matrix Science) against the UniProt human database.

### 2.10 Statistical analysis

The analysis of results was performed using GraphPad Prism (GraphPad Software). All data were presented as mean ± SD except where indicated otherwise. All comparisons were carried out using the Student t test to assess the significance of the results unless otherwise specified. Statistical significance was established at *P* < .05.

## 3 RESULTS

### 3.1 *HN252 inhibits the activity of PPM1B* in vitro

**Figure 1:**
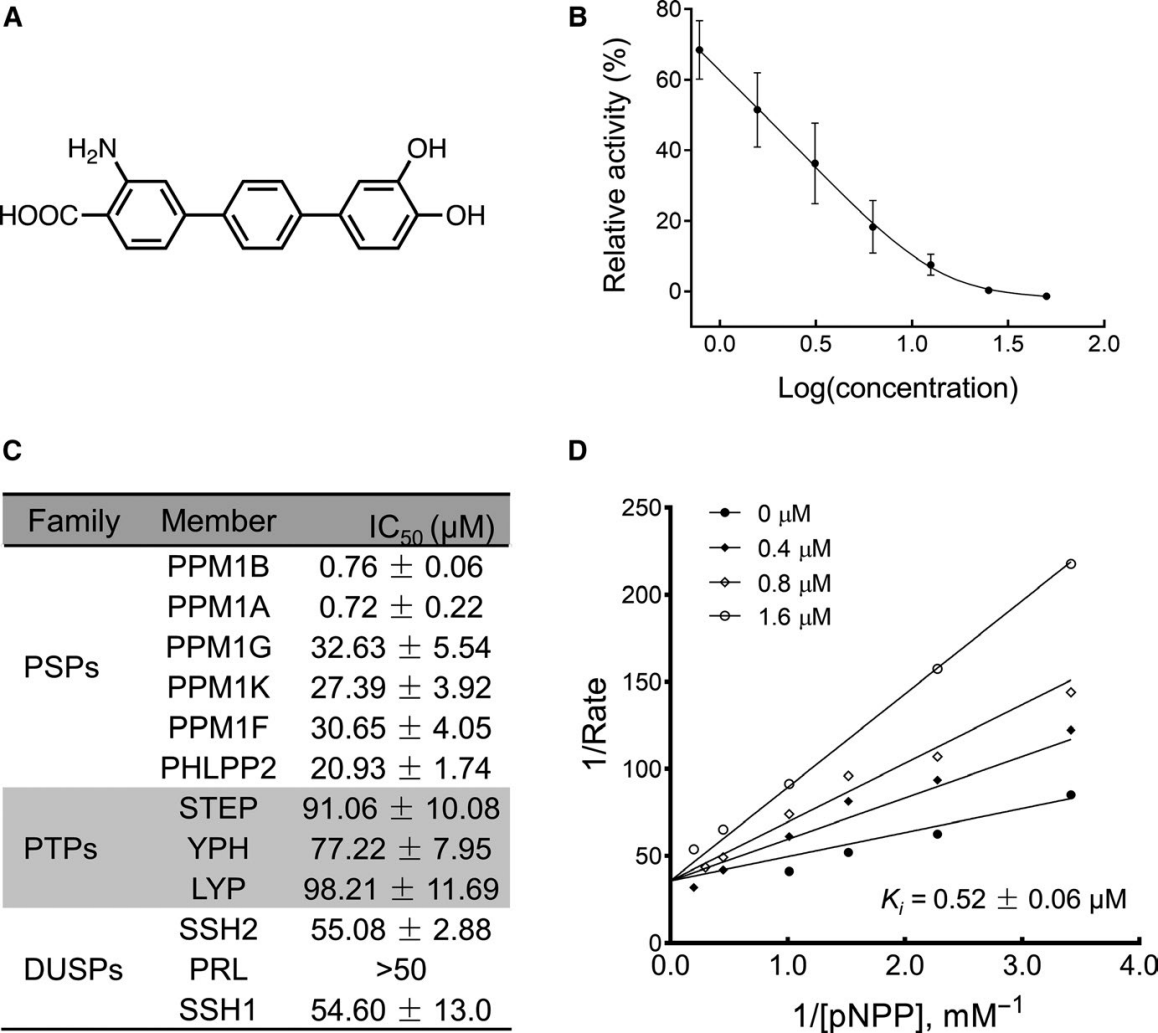
HN252 is a potent inhibitor of PPM1B. (A) Chemical structure of HN252. (B) Effect of HN252 on the PPM1B (0.4 μM PPM1B with 40 mM Mn^2+^)‐catalysed hydrolysis of p‐nitrophenyl phosphate (pNPP) at pH 7.0, 37°C. Data were obtained from three independent experiments and shown as mean ± SD. (C) Selectivity of HN252 towards a panel of protein phosphatases. All measurements were carried out using pNPP as a substrate. The IC_50_ values were obtained from three independent experiments and shown as mean ± SD. PSPs: protein serine/threonine phosphatases; PTPs: protein tyrosine phosphatases; DUSPs: dual‐specificity phosphatase. (D) The kinetic profile of HN252 against PPM1B. Lineweaver‐Burk plot showing the effect of HN252 on the PPM1B‐catalysed hydrolysis of pNPP at pH 7.0, 37°C, which indicates competitive inhibition. PPM1B is at 0.4 μM with 40 mM Mn^2+^. pNPP concentrations were 0.29, 0.43, 0.65, 0.98, 1.48, 2.22, 3.33 and 5 mM, respectively. *K_i_* was obtained from three independent experiments and shown as mean ± SD

To identify the inhibitors of PPM1B, we used a p‐nitrophenyl phosphate (pNPP) hydrolysis‐based phosphatase in vitro assay to screen a library containing natural products and their derivatives.^18^ These compounds were previously designed to develop inhibitors of topoisomerases which are mainly based on the ATP/ADP binding inhibition.^16^, ^25^, ^26^, ^27^, ^28^ As protein phosphatases are involved in the transfer of phosphate groups, we speculate that these compounds could present inhibitory activity against PPM1B. Several hits were identified to show dramatical decrease in pNPP hydrolysis, which correlates with the inhibition of phosphatase activity of PPM1B (Figure S1). HN252, a p‐terphenyl derivative, exhibited potent inhibitor activity against PPM1B in a dose‐dependent manner (Figure 1A and B). Although it is structurally similar to the identified topoisomerase inhibitors, HN252 presents no inhibition against topoisomerases in vitro and also no detectable cytotoxicity in our study in the low micromolar range (data not shown).

To determine the selectivity of HN252 towards PPM1B, we evaluated its inhibitory activity against a panel of protein phosphates including STEP, YPH, LYP, SSH1, SSH2 and PRL. Strikingly, under identical assay conditions, HN252 specifically inhibited the activity of PPM1B with an IC_50_ of 0.76 µM which is > 70‐fold lower than that of other phosphatases tested (Figure 1C). Moreover, HN252 showed about 30‐fold increase in potency against PPM1B than other PSPs (eg PPM1A, PPM1F, PPM1G, PPM1K and PHLPP2) with PPM1A as a notable exception (Figure 1C). Although PPM1A and PPM1B proteins share highly conserved amino acid sequence,^29^ the enzyme kinetic profile of HN252 showed it to be a classical competitive inhibitor against PPM1B (*K_i_* = 0.52 ± 0.06 µM) (Figure 1D), whereas it is a non‐competitive inhibitor against PPM1A (Figure S2).

### 3.2 Direct interaction of HN252 with PPM1B leads to inhibition of PPM1B activity

**Figure 2:**
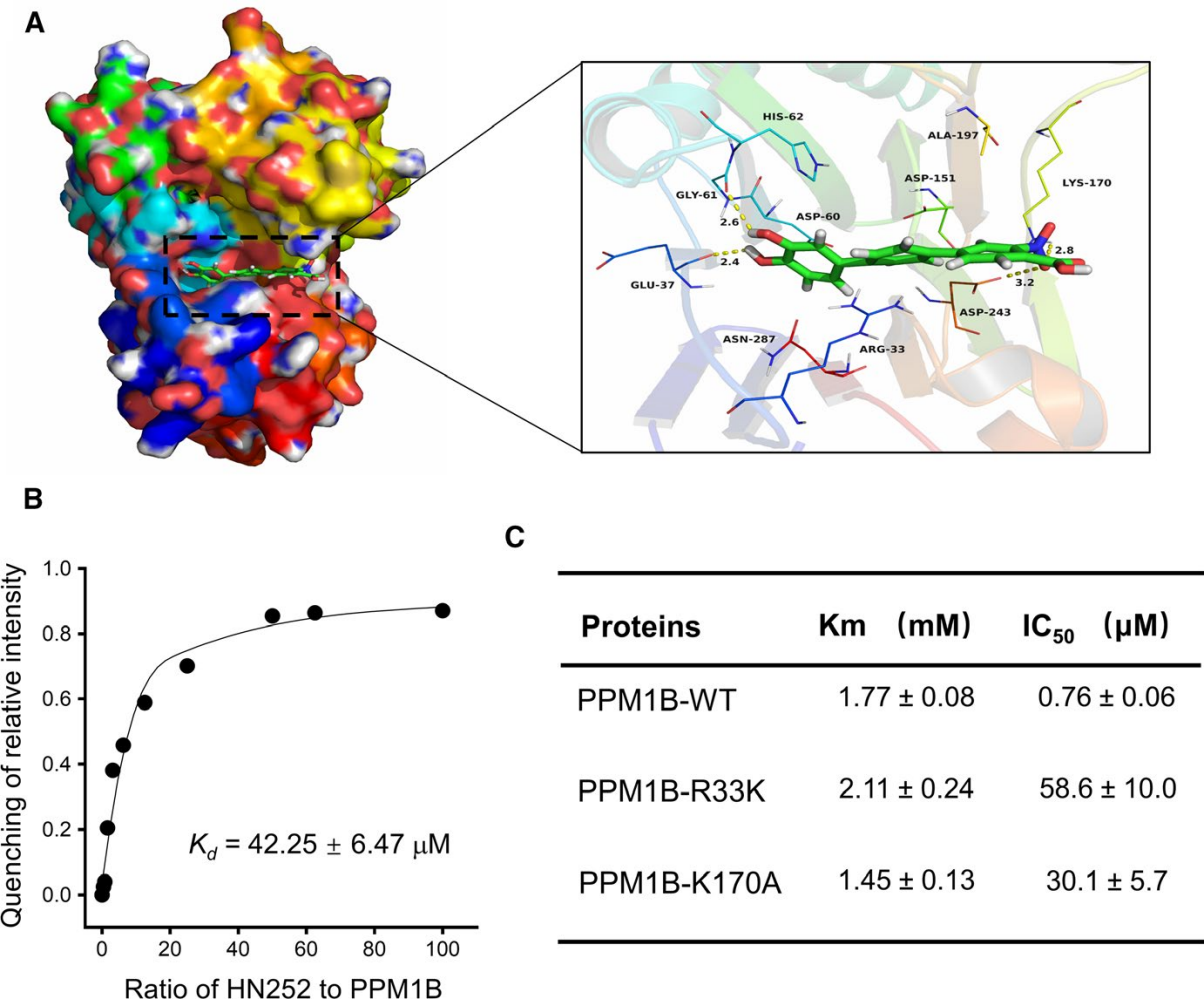
HN252 binds to PPM1B. (A) 3D (left) and surface (right) view models show the binding pocket and binding of PPM1B and HN252. Molecular docking of HN252 with PPM1B (PDB: 2P8E) was performed. (B) The change in fluorescence intensity of PPM1B upon increasing concentration of HN252. The plot indicated the titration of intensity of PPM1B (8 μM) with HN252 against PPM1B single at *λ*_em_ = 332 nm. HN252 concentrations were 0, 3.125, 6.25, 12.50, 25, 50, 100, 200, 400, 500 and 600 μM, respectively. The dissociation constant (*K_d_*) is obtained from three independent experiments and shown as mean ± SD. (C) HN252 towards inhibition of PPM1B mutants. All measurements were carried out using pNPP as a substrate at pH 7.0, 37°C. The *Km* and IC_50_ values were obtained from three independent experiments and shown as mean ± SD

To reveal the molecular basis of the inhibitory activity, we performed molecular docking study with 100‐ns molecular dynamics simulations. The root‐mean‐square deviation (RMSD) values of the protein backbone based on the starting structure along the simulation time were calculated, which indicated that PPM1B‐HN252 complex was stabilized during the 100‐ns simulation (Figure S3A). Compound HN252 adopted a compact conformation to bind in the site of the PPM1B (Figure 2A). The cation‐π interaction was observed between the compound HN252 and the residue Arg‐33. Detailed analysis showed that the phenyl groups of the HN252 formed anion‐π interactions with the residues Asp‐60, Asp‐151 and Asp‐243, respectively. Importantly, four hydrogen bond interactions were observed between the HN252 and the residues Glu‐37 (bond length: 2.4 Å), Gly‐61 (bond length: 2.6 Å), Lys‐170 (bond length: 2.8 Å) and Asp‐243 (bond length: 3.2 Å), which was the main interaction between the HN252 and the PPM1B. The binding is directly confirmed by the internal fluorescence quenching measurement assay, which revealed real biophysical association between HN252 and PPM1B with a *K_d_* value of 42.25 ± 6.47 µM (Figure 2B).

We then made a series of PPM1B mutants to confirm the key protein residues responsible for the binding of HN252 to PPM1B. The mutations Q37A, D60A and G61A totally abolished the activity of PPM1B‐catalysed hydrolysis of pNPP (data not shown), suggesting that these protein residues are critical for the binding of subtracts. Compared to the wild‐type enzyme, the mutants R33K and K170A displayed comparable *K_m_* values but showed notably decreased response to HN252 treatment (Figure 2C), indicating that these two mutations impaired the affinity of HN252 on the PPM1B. Additionally, the binding energy of HN252 to PPM1B‐R33K and PPM1B‐K170A was distinctly reduced compared to that of HN252 and PPM1B (Figure S3B), which further confirmed that these two residues significantly contribute to the binding of HN252 to PPM1B.

### 3.3 HN252 inhibits the intracellular PPM1B activity

**Figure 3:**
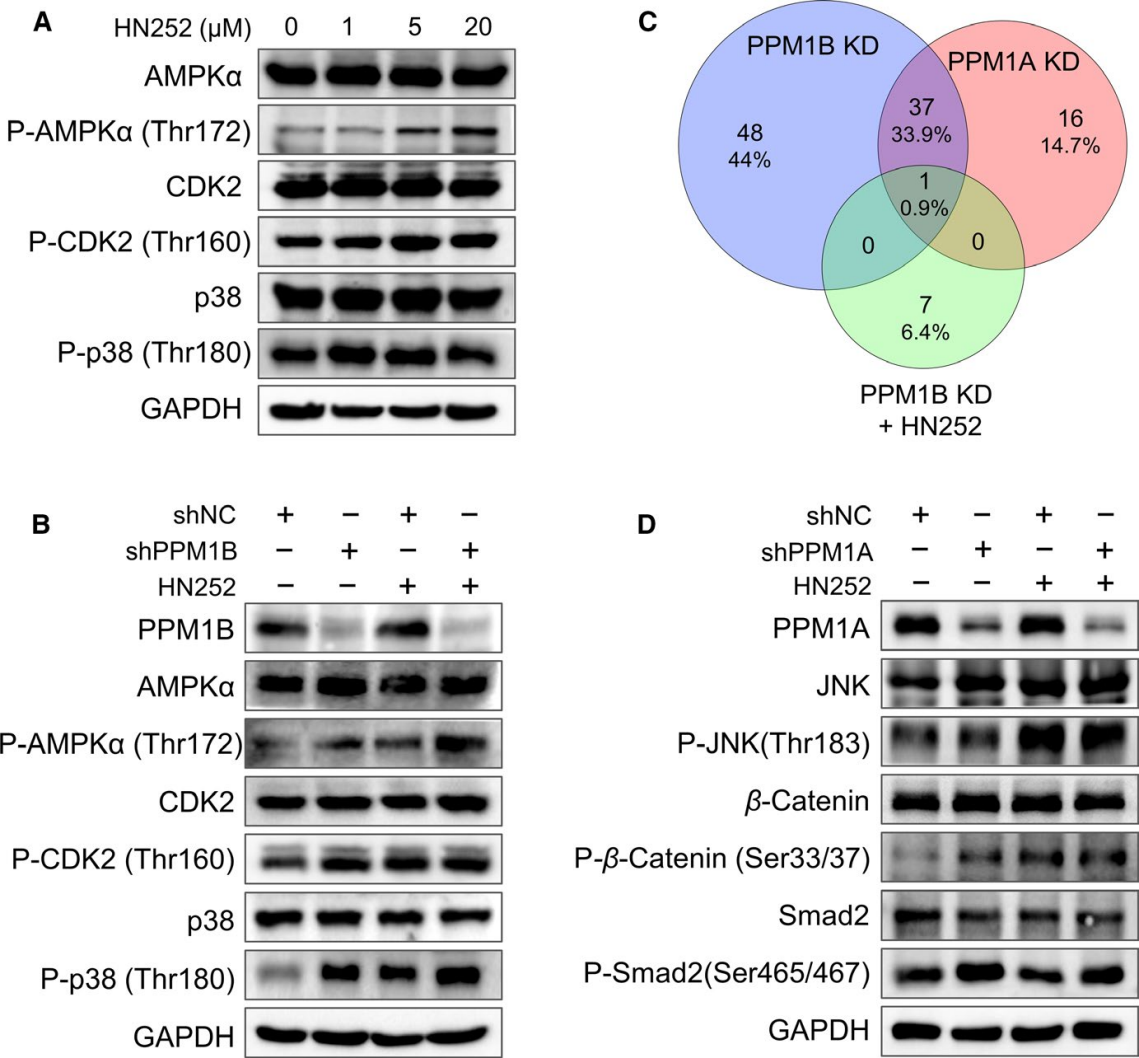
HN252 inhibits the intracellular PPM1B activity. (A) Western blot analysis of indicated protein expression and their phosphorylation in HL7702 cells with HN252 treatment. Increasing concentration of HN252 was added to HL7702 cells for 2 h. GAPDH was used as a loading control. An equal number of cells were loaded in each well. (B) Western blot analysis of indicated protein expression and their phosphorylation in HL7702 cells after knockdown of PPM1B in the presence of HN252. HL7702 cells were transduced by lentiviruses encoding indicated shRNA and then were treated with HN252 (5 μM) for 2 h at 30 h after transfection. shNC represents a non‐targeting shRNA targeting scramble. GAPDH was used as a loading control. An equal number of cells were loaded in each well. (C) Venn diagram indicated the number of elevated protein phosphorylation (>1.5‐fold up‐regulation) from PPM1B knockdown, PPM1B knockdown plus HN252 treatment and PPM1A knockdown. The total lists of this protein phosphorylation are shown in Tables S1 and S2, respectively. (D) Western blot analysis of indicated protein expression and their phosphorylation in HL7702 cells after knockdown of PPM1A in the presence of HN252. HL7702 cells were transduced by retroviruses encoding PPM1A shRNA and then were treated with HN252 (5 μM) for 2 h at 30 h after transfection. shNC represents a non‐targeting shRNA targeting scramble. GAPDH was used as a loading control. An equal number of cells were loaded in each well

It has been demonstrated that PPM1B is involved in the dephosphorylation of several proteins^10^, ^14^, ^30^, ^31^; we therefore investigated the effect of HN252 on the phosphorylation of these physiological substrates in cellular context. As expected, the phosphorylation of AMPKα, CDK2 and p38 was dramatically increased in HL7702 cells with HN252 treatment (Figure 3A). Similar up‐regulation of proteins phosphorylation was also observed in HCT116, PC‐3 and COS7 cells in the presence of HN252 (Figure S4).

To further confirm the effect of HN252 on the PPM1B, HL7702 cells were transduced with PPM1B shRNA encoded by lentivirus and then treated with HN252. The knockdown of PPM1B led to enhanced phosphorylation of AMPKα, CDK2 and p38 in HL7702 cells; however, HN252 treatment had a mild effect on the phosphorylation of these proteins in HL7702 cells with PPM1B shRNA (Figure 3B). Furthermore, a Phospho Explorer Antibody Microarray was performed to examine the phosphorylation profiling of broad‐scope proteins. A total of 86 proteins were identified, whose phosphorylation on serine/threonine sites was greatly up‐regulated after PPM1B knockdown (>1.5‐fold vs scramble shRNA). However, only 8 proteins with further enhanced phosphorylation were detected in the presence of HN252 compared to PPM1B knockdown (>1.5‐fold) (Figure 3C and Table S1).

Given the comparable inhibitory activity of HN252 against PPM1B and PPM1A in vitro phosphatase assay, we dissected it effect on the intracellular PPM1A. Fifty‐four proteins’ phosphorylation was dramatically up‐regulated after PPM1A knockdown, and most of them were also observed in PPM1B knockdown cells. This is agreement with that PPM1B and PPM1A shared similar physiological substrates.^30^ However, the elevated protein phosphorylation in PPM1B knockdown cells with HN252 treatment was not found in the PPM1A knockdown cells (Figure 3C and Table [Link], [Link]). Consistently, enhanced phosphorylation of β‐catenin (one of common protein phosphorylation up‐regulated by PPM1B and PPM1A knockdown) was confirmed by immunoblotting in both PPM1A knockdown cells and HN252‐treated cells, whereas the phosphorylation of JNK up‐regulated by PPM1B knockdown is only up‐regulated in HN252‐treated cells but not in the PPM1A knockdown cells (Figure 3D and Table S1). Furthermore, smad2 (reported substrate for PPM1A) was hyperphosphorylated after PPM1A knockdown but not PPM1B knockdown and HN252 treatment (Figure 3D and Table S1). These data indicated that HN252 mainly inhibits the intracellular activity of PPM1B.

### 3.4 PPM1B inhibition to identify the relative substrates and cellular processes

**Figure 4:**
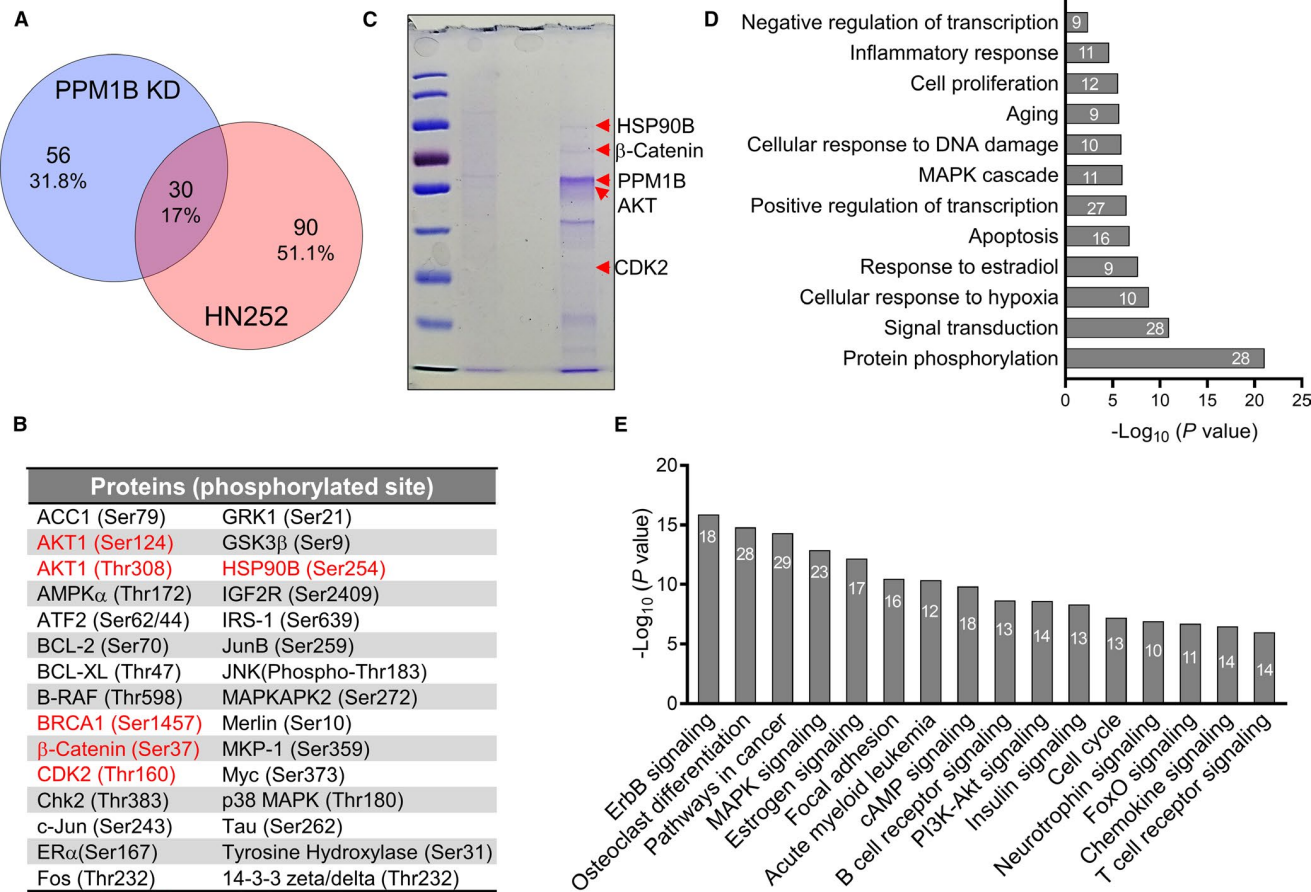
Identification of novel substrates of PPM1B. (A) Venn diagram indicated the number of elevated protein phosphorylation (>1.5‐fold up‐regulation) from PPM1B knockdown and HN252 treatment, respectively. The total lists of this protein phosphorylation are shown in Tables S1 and S5, respectively. (B) The overlapped protein phosphorylation from PPM1B knockdown and HN252 treatment as shown in panel A. Red indicates PPM1B interacting partners identified by immunoprecipitation followed by mass spectrometry (IP‐MS) in panel C. (C) Coomassie blue staining of HA immunoprecipitation (IP) in HL7702 cells overexpressed HA‐PPM1B and blank vector, respectively. Arrow indicates predicted size of PPM1B, HSP90B, β‐catenin, AKT and CDK2. The total list of PPM1B interacting partners is shown in Table S4. (D–E) Representative biological processes (D) and signalling pathways (E) significantly enriched from proteins differentially regulated by HN252 treatment. The proteins (>1.5‐fold increased phosphorylation shown in Table S5 for 2 h treatment) were mapped to GO and KEGG analysis using DAVID bioinformatics database. Numbers shown in each bar indicate the number of enriched proteins in relative terms. The total list of enriched GO and KEGG terms is shown in Table S6 with enrichment ratio > 10% and enrichment *P* value < 0.01

We then aimed to identify potential substrates that can be dephosphorylated by PPM1B. To this end, we performed phosphorylation profiling of broad‐scope proteins in HL7702 with HN252 treatment using a Phospho Explorer Antibody Microarray. The global changes of proteins phosphorylation are dynamics during 14 hours in the presence of HN252 (Figure S5 and Table S5), which is consistent with the profiling of phosphatases that is the highly transient interaction between most phosphatases and their substrates and the poor substrate specificity.^32^ Given the above dynamics of phosphorylation, we mainly focus on the differentially regulated protein phosphorylation after 2‐hours exposure. A wide range of proteins with enhanced phosphorylation was identified (1.5‐fold vs control), 30 of which were also elevated in PPM1B knockdown cells (Figure 4A and B), suggesting these proteins are the potential substrates of PPM1B. Indeed, previously reported substrates such as AMPKα, p38 and CDK2 are also included, which increased our confidence in identifying novel substrates for PPM1B.

We next performed immunoprecipitation followed by mass spectrometry (IP‐MS) in HL7702 cells overexpressed HA‐PPM1B or blank vector control (Figure 4C). A total of 132 proteins were identified that were specifically detected in IP from cells overexpressed HA‐PPM1B compared to that of blank vector control cells (Table S4). Among these PPM1B interacting partners, 5 relative substrates were demonstrated as they were strongly phosphorylated (>1.5‐fold increase) after PPM1B knockdown and HN252 treatment, respectively, including one known (CDK2) and 4 novel ones (AKT1, HSP90B, β‐catenin and BRCA1) (Figure 4B).

To better understand the global roles of PPM1B in the cellular processes, we mapped the differentially regulated proteins (>1.5‐fold increased phosphorylation) to the enrichment analysis after the PPM1B knockdown and HN252 treatment, respectively (Table S6‐7).^33^ Venn diagram revealed that these two groups showed similar enrichments of biological processes and signalling pathways, even they shared less common regulated protein phosphorylation (Figure 4A and Figure S6A‐B). Those regulated proteins were highly enriched in fundamental processes involved in protein phosphorylation, apoptosis, gene transcription, ageing and cell proliferation (Figure 4D and Figure S6C). GO term enrichment analysis indicated that PPM1B is also related to inflammatory regulation. This is in agreement with KEGG analysis, which identified relative signalling pathways, such as chemokine signalling, B‐/T‐cell receptor signalling and neurotrophic signalling (Figure 4E and Figure S6D).

In addition, these proteins regulated by PPM1B inhibition were notably concentrated in multiple signalling pathways besides previously reported ones, including PI3K/AKT signalling, cAMP signalling, ErbB signalling, FOXO signalling and insulin signalling. Moreover, pathways in solid tumours and leukaemia were also appeared in our enrichment analysis, indicating that PPM1B plays roles in tumorigenesis (Figure 4E).

## 4 DISCUSSION

Although genetic and biochemical strategies such as knockdown and knockout have widely contributed to the understanding of protein functions, such methods have limitations in informing on rapid kinetic changes of phosphatases, elucidating functional effects of specific phosphorylation events. Compared to these methods, small‐molecule modulators are an ideal tool for understanding of their mechanisms, substrates and exclusive functions within highly intricate networks.^34^ In this study, we demonstrated that HN252 is a novel potent inhibitor of PPM1B, by which we identified several potential substrates and relative cellular processes regulated by PPM1B.

PPM1B and PPM1A shared similar amino acid sequences and exhibited a comparable substrate preference.^30^ The enzyme kinetic profile of HN252 proved it as a classical competitive inhibitor against PPM1B but a non‐competitive inhibitor against PPM1A. Although we cannot absolutely eliminate the co‐effect of PPM1A inhibition on the enhanced protein phosphorylation in cells, our data indicated that HN252 mainly inhibits the intracellular activity of PPM1B (Figure 3C,D). This is further demonstrated by the inefficiency of HN252 on the PPM1A‐specific substrates in HL7702 cells. Our findings suggest that HN252 is an ideal starting point to develop specific inhibitors for PPM1B and PPM1A separately based upon differences in binding site residues.

The identification of phosphatase substrate is challenging to address because of highly transient interaction. Given the change in the phosphorylation status reflects an altered balance between both involved kinases and phosphatases,^35^ phosphatase inhibition can lead to the hyperphosphorylation of their relative substrates. Indeed, our data showed that both HN252 treatment and PPM1B knockdown up‐regulated many proteins’ phosphorylation in HL7702 cells (Figure 3 and Figure 4B,C). From this viewpoint, global phosphorylation assay would be a powerful strategy for high‐throughput cell‐based substrates screening. Accordingly, we successfully identified 30 novel potential substrates overlapped from PPM1B knockdown and HN252 treatment (Figure 4A,B). Among these candidates, five relative substrates were further confirmed by immunoprecipitation, including one known and 4 novel ones. However, HN252 treatment and PPM1B knockdown also down‐regulated many proteins’ phosphorylation in HL7702 cells, which may be resulted from the enhanced protein phosphorylation. Furthermore, we cannot eliminate the possibility that enhanced protein phosphorylation is indirectly resulted from PPM1B inhibition. Therefore, newly identified candidates require further confirmation of their physiological interactions with PPM1B by the aid of novel technologies such as bimolecular fluorescence complementation (BiFC)^36^ and FRET‐FLIM.^37^

As phosphatases counteract kinases that had established oncogenes and drug targets for targeted cancer therapy, they have traditionally been assumed to be tumour suppressors. However, some phosphatases have been reported to function as both tumour suppressors and oncogenes.^8^, ^38^ Because of the limited understanding of its physiological functions, the biological relevance of PPM1B in the human cancers is still unclear. Our data indicated that PPM1B is involved in the pathways in multiple cancers including leukaemia (Figure 4E and Figure S6D). Indeed, the expression of PPM1B was markedly reduced in diverse cancers and specifically in leukaemia cells (Figure S7), indicating that PPM1B is a tumour suppressor. As a further evidence, PPM1B was demonstrated to negatively regulate cancer cell motility and invasiveness through inhibition of Rho GTPase activity.^39^

GO and KEGG enrichments revealed that PPM1B activity is also correlated to many cellular processes and signalling pathways, such as gene transcription. Consistently, recent work demonstrated that PPM1B is involved in pre‐mRNA synthesis through the interaction with hMTr1.^40^

In summary, we identified HN252 as a novel PPM1B inhibitor and dissected the intracellular relevance of PPM1B inhibition. Our findings provide novel insights into the understanding of the physiological functions of PPM1B.

## CONFLICT OF INTEREST

The authors declare that they have no conflicts of interest with the contents of this article.

## AUTHOR CONTRIBUTIONS

**Zhiyuan Lu:** Investigation (lead). **Peng Xiao:** Investigation (equal). **Yuan Zhou:** Investigation (equal). **Zhenyu Li:** Investigation (equal). **Xiao Yu:** Methodology (equal). **Jinpeng Sun:** Methodology (equal); Project administration (equal). **Yuemao Shen:** Methodology (equal); Project administration (equal); Writing‐review & editing (equal). **Baobing Zhao:** Methodology (lead); Project administration (lead); Writing‐original draft (lead); Writing‐review & editing (lead).

## Supporting information

Figure S1

Figure S2

Figure S3

Figure S4

Figure S5

Figure S6

Figure S7

Table S1

Table S2

Table S3

Table S4

Table S5

Table S6

Table S7

Table S8

Supplementary Material
