# Supplementary figures and images for: Identification of HN252 as a potent inhibitor of protein phosphatase PPM1B
Source: J Cell Mol Med. 2020 Oct 13;24(22):13463–71. doi: 10.1111/jcmm.15975 (PMC7701510; doi:10.1111/jcmm.15975)

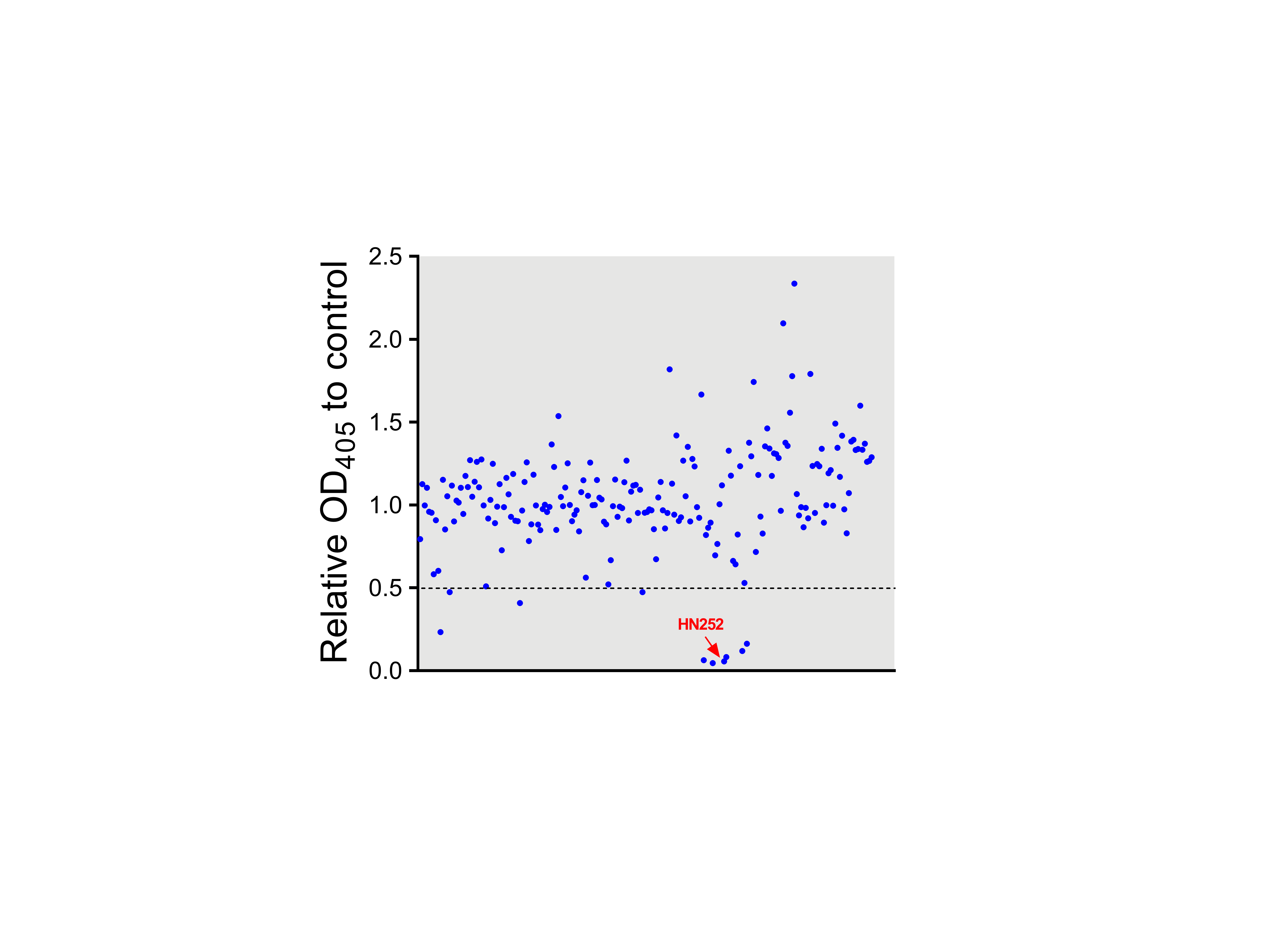

Supplement: Supplementary file 1 — Figure S1 [file JCMM-24-13463-s001.TIF]

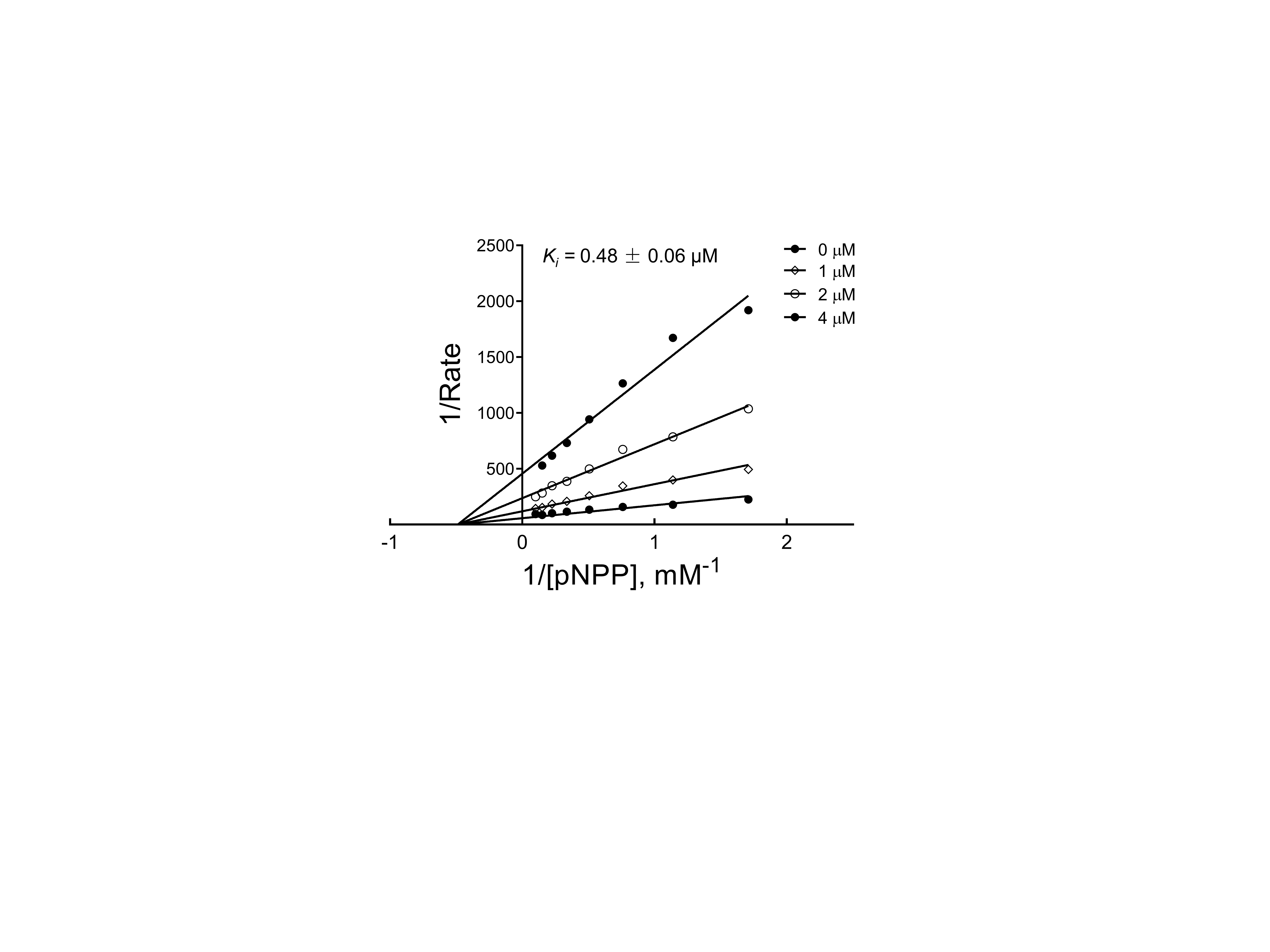

Supplement: Supplementary file 2 — Figure S2 [file JCMM-24-13463-s002.TIF]

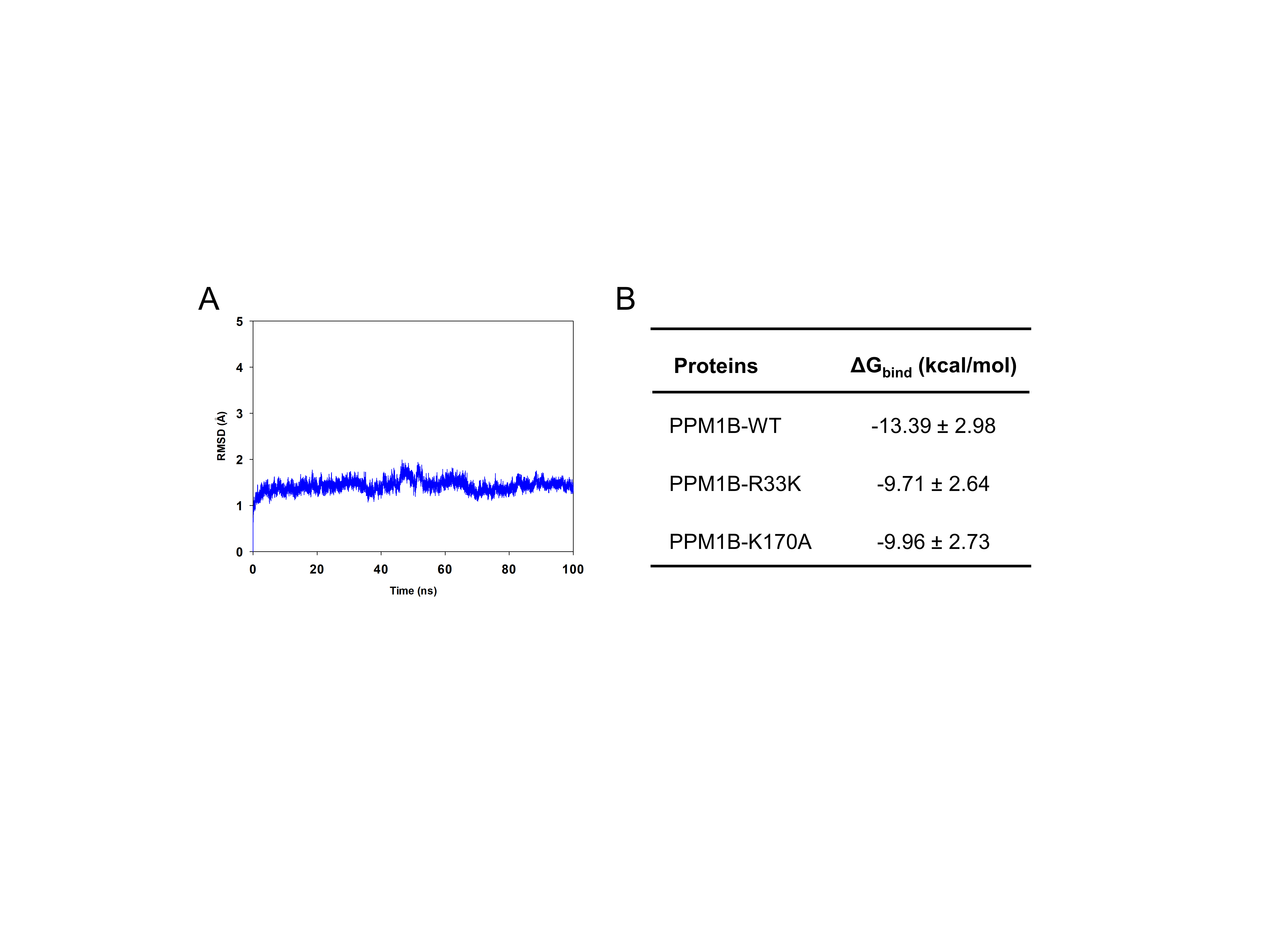

Supplement: Supplementary file 3 — Figure S3 [file JCMM-24-13463-s003.TIF]

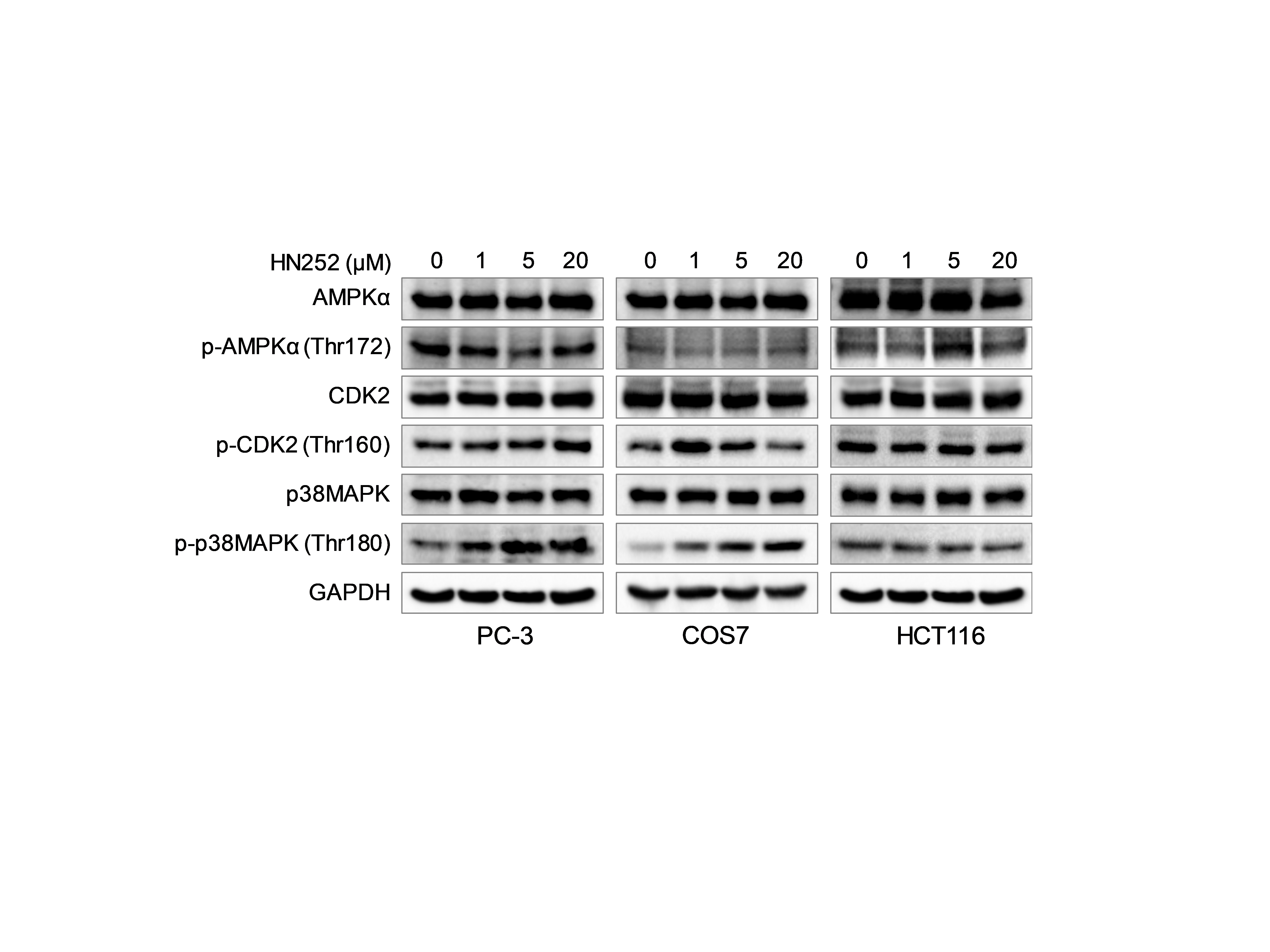

Supplement: Supplementary file 4 — Figure S4 [file JCMM-24-13463-s004.TIF]

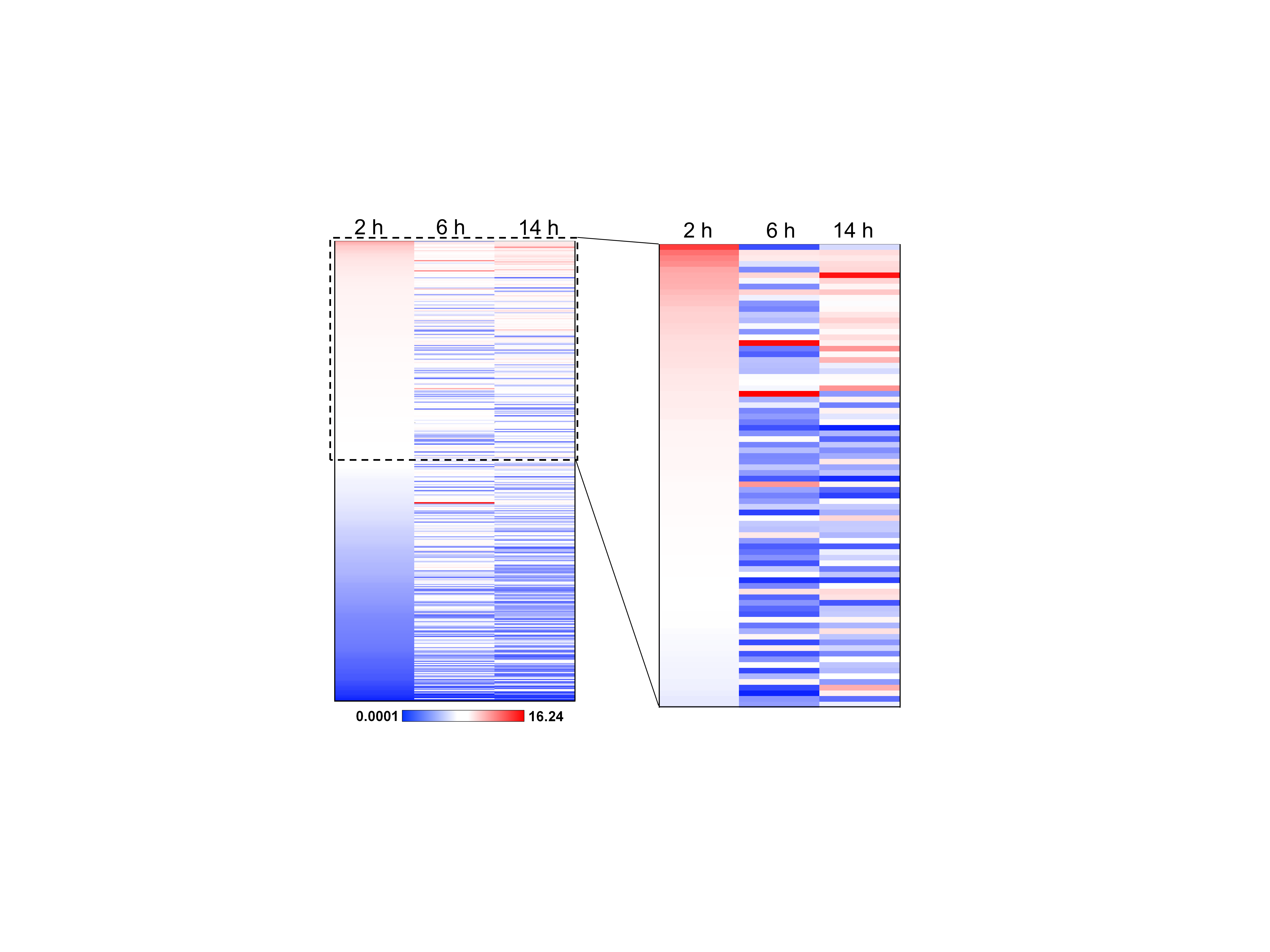

Supplement: Supplementary file 5 — Figure S5 [file JCMM-24-13463-s005.TIF]

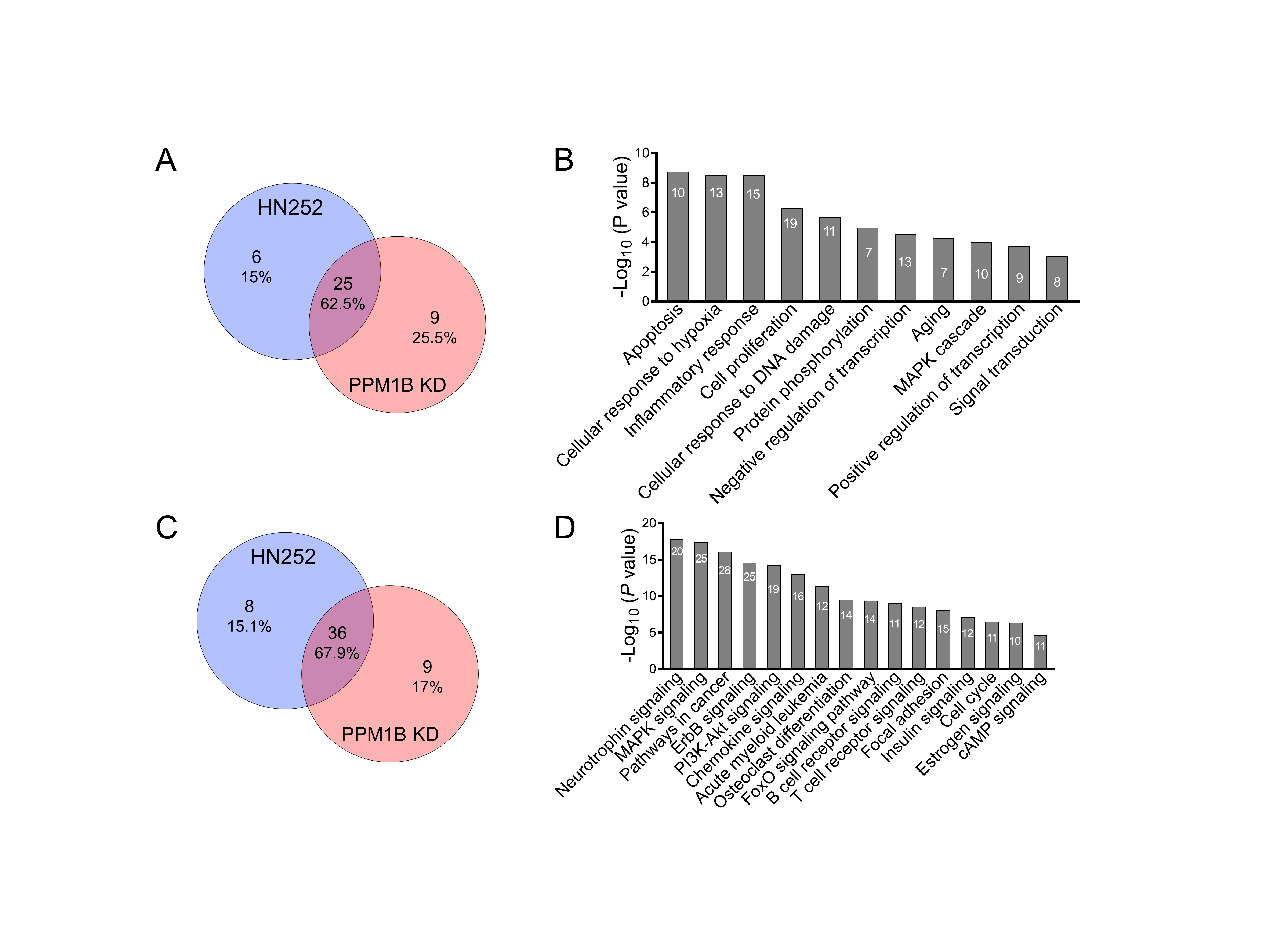

Supplement: Supplementary file 6 — Figure S6 [file JCMM-24-13463-s006.TIF]

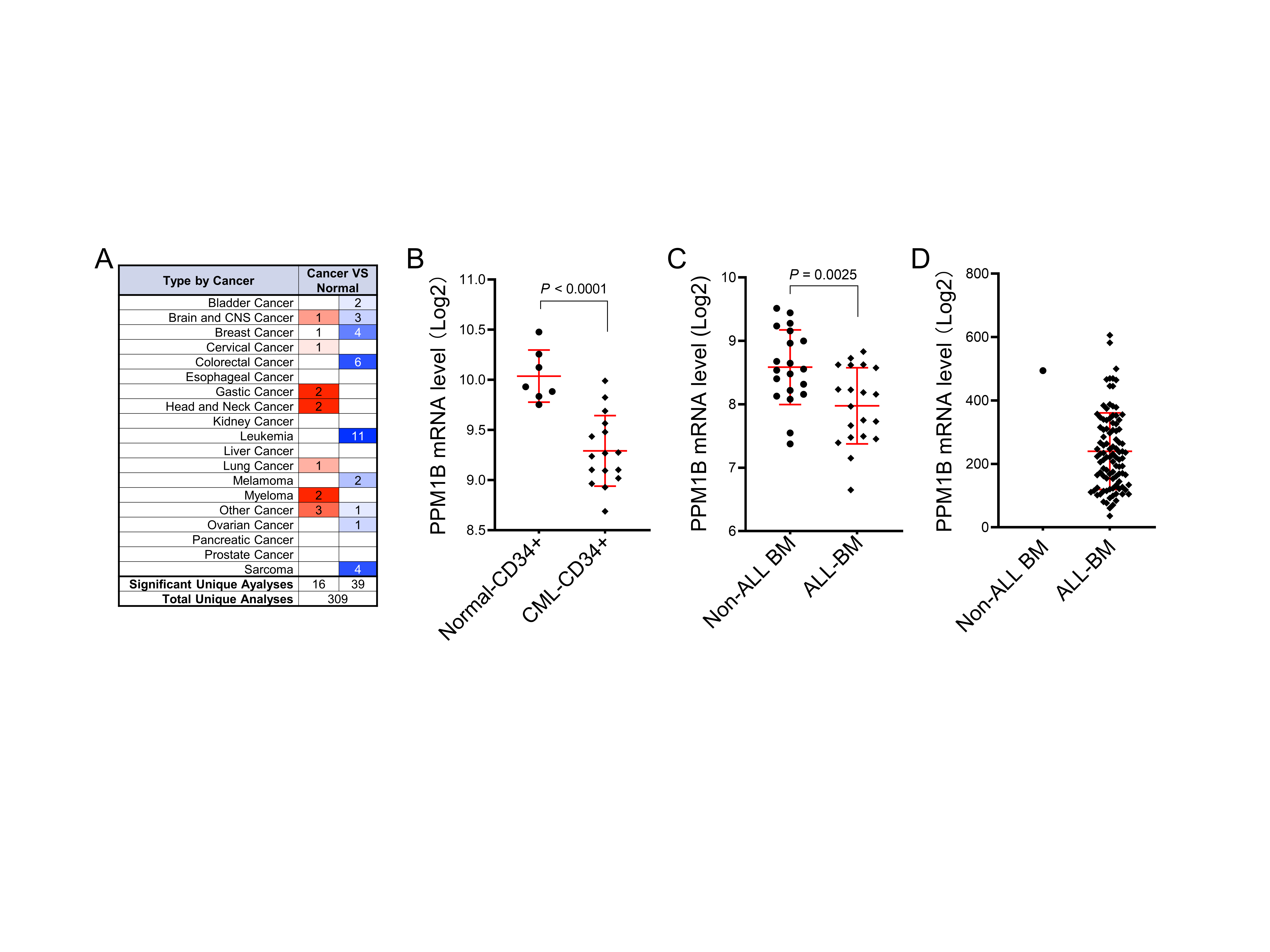

Supplement: Supplementary file 7 — Figure S7 [file JCMM-24-13463-s007.TIF]
