# Supplementary material for: Identification of HN252 as a potent inhibitor of protein phosphatase PPM1B
Source: J Cell Mol Med. 2020 Oct 13;24(22):13463–71. doi: 10.1111/jcmm.15975 (PMC7701510; doi:10.1111/jcmm.15975)
Supplement: Supplementary file 15 — Table S8 [file JCMM-24-13463-s015.docx]

**Table S8 Antibodies and commercial reagents used in this study**

| Antibody | Source | Catalog Number |
| --- | --- | --- |
| Anti-AMPKα | CST | 5831 |
| Anti-phospho-AMPKα(Thr172) | CST | 2535 |
| Anti-CDK2 | ProteinTech | 10122-1-AP |
| Anti-phospho-CDK2(Thr160) | Abcam | ab194868 |
| Anti-p38 MAPK | ProteinTech | 14064-1-AP |
| Anti-phospho-p38 MAPK(Thr180) | Abcam | ab178867 |
| Anti-PPM1B | Abcam | ab70804 |
| Anti-β-catenin | CST | 8480 |
| Anti-phosphor-β-catenin (Ser33/37Thr41) | CST | 9561 |
| Anti-JNK | ProteinTech | 51151-1-AP |
| Anti-phosphor-JNK(Thr183) | CST | 9251 |
| Anti-Smad2 | CST | 5339 |
| Anti-Phospho-Smad2 (Ser465/467) | CST | 18338 |
| Anti-PPM1A | Abcam | Ab154489 |
| Anti-GAPDH | Abcam | ab9385 |
| Goat Anti-Rabbit IgG (H+L) | Jackson | 111-035-003 |
| Goat Anti-Mouse IgG (H+L) | Jackson | 115-035-003 |
| Phospho Explorer Antibody Array | Full Moon Biosystem | PEX100 |
| LipoFiterTM reagent | Hanbio | HB-TRLF |
| cOmplete™ Protease Inhibitor Cocktail | Roche | 4693116001 |
| PhosSTOP™ | Roche | 4906845001 |
| Ni-NTA Agarose | Qiagen | 30210 |
| Anti-HA Magnetic Beads | Thermo Scientific | 88836 |
