# Supplementary material for: Identification of HN252 as a potent inhibitor of protein phosphatase PPM1B
Source: J Cell Mol Med. 2020 Oct 13;24(22):13463–71. doi: 10.1111/jcmm.15975 (PMC7701510; doi:10.1111/jcmm.15975)
Supplement: Supplementary file 16 — Supplementary Material [file JCMM-24-13463-s016.docx]

**Supplemental Information**

**Identification of HN252 as a potent inhibitor of protein phosphatase PPM1B**

Zhiyuan Lu^1#^, Peng Xiao^2#^, Yuan Zhou^1^, Zhenyu Li^4^, Xiao Yu^2^, Jinpeng Sun^2^, Yuemao Shen^1^, Baobing Zhao^1,3*^

^1^Key Laboratory of Chemical Biology (Ministry of Education), School of Pharmaceutical Sciences, Cheeloo College of Medicine, Shandong University, Jinan, China, 250012;

^2^Key Laboratory Experimental Teratology of the Ministry of Education and Department of Biochemistry and Molecular Biology, School of Medicine, Cheeloo College of Medicine, Shandong University, Jinan, China 250012;

^3^Department of Pharmacology, School of Pharmaceutical Sciences, Cheeloo College of Medicine, Shandong University, Jinan, China, 250012;

^4^Department of Pharmacy, Shandong Provincial Hospital, Cheeloo College of Medicine, Shandong University, Jinan, China, 250012.

**Supplemental Figure legends**

**Figure S1. Screening of inhibitor against PPM1B in vitro**. Scatter plot showing the OD_405_ normalized to DMSO control. Each dot represents an individual compound. The dotted line indicates 50% of the control. HN252 was highlighted in red color.

**Figure S2. The kinetic profile of HN252 against PPM1A.** Lineweaver-Burk plot showing the effect of HN252 on the PPM1A-catalyzed hydrolysis of pNPP at pH 7.0, 37℃, which indicates non-competitive inhibition. PPM1A is at 0.4 μM with 40 mM Mn^2+^. pNPP concentrations were 0.58, 0.87, 1.31, 1.97, 2.96, 4.44, 6.66 and 10 mM, respectively. *K_i_* was obtained from three independent experiments and shown as mean ± SD.

**Figure S3. MD refinement of the PPM1B-HN252 complex.** (**A**) The root-mean-square deviations (RMSDs) of all the atoms of PPM1B-HN252 complex with respect to its initial structure as function of time. (**B**) The binding free energies between PPM1Bs and HN252. The binding free energies (ΔG_bind_ in kcal/mol) were calculated using the Molecular Mechanics/Generalized Born Surface Area (MM/GBSA) method, implemented in AmberTools 13.

**Figure S4. HN252 inhibits the intracellular PPM1B activity.** Western blot analysis of indicated proteins expression and their phosphorylation in cultured cells with HN252 treatment. Different concentrations of HN252 were added to the cultured cells for 2 h. GAPDH was used as a loading control. An equal number of cells were loaded in each well.

**Figure S5. Heatmap illustrating the differentially regulated phosphorylation of broad-scope proteins in HL7702 with HN252 treatment.**Enlarged heatmap is shown on the left illustrating proteins phosphorylation with over 1.5 -fold upregulation after 2 h exposure. HL7702 cells were treated with either HN252 (5 μM) or vehicle for indicated times and harvested for analysis of phosphorylation profiling of broad-scope proteins by using a Phospho-Explorer Antibody Microarray. The color indicated the fold changes of protein phosphorylation normalized to that of vehicle control after HN252 treatment.

**Figure S6. GO and KEGG enrichment analysis of hyperphosphorylated proteins after PPM1B knockdown.** The proteins (>1.5-fold increased phosphorylation shown in Table S6) were mapped to GO and KEGG analysis using DAVID bioinformatics database. (**A**) Venn diagram revealed the similar enrichments of GO (biological process) terms from PPM1B knockdown and HN252 treatment respectively. (**B**) Representative biological processes significantly enriched from proteins differentially regulated by PPM1B knockdown. Numbers shown in each bar indicate the number of enriched proteins in relative terms. (**C**) Venn diagram revealed the similar enrichments of KEGG pathways from PPM1B knockdown and HN252 treatment respectively. (**D**) Representative signaling pathways significantly enriched from proteins differentially regulated by PPM1B knockdown. Numbers shown in each bar indicate the number of enriched proteins in relative terms. The total list of enriched GO and KEGG terms is shown in Table S7.

**Figure S7. The expression of PPM1B in cancers.** (**A**) Gene expression of PPM1B in 15119 samples derived from diverse cancers. Data were obtained from Oncomine database ([www.oncomine.org](http://www.oncomine.org/)). Number in cells mean the analyses shown significantly altered expression, and the color indicates the gain (red) or loss (blue) of expression of PPM1B. P < 0.001, fold change > 1.5. (**B-D**) Gene expression levels of PPM1B in samples derived from leukemia patients and their corresponding normal controls. Data were obtained from GSE24739 (**B**, chronic myelogenous leukemia), GSE17703 (**C**, acute lymphocytic leukemia) and GSE14858 (**D**, acute myeloid leukemia). *P* value was determined by 2-tailed t test.

**Supplemental Table legends**

**Table S1.** The phosphorylation profiling of broad-scope proteins in HL7702 transduced with PPM1B shRNA with or without HN252 treatment for 1 hour. Red indicated the elevated protein phosphorylation (>1.5-fold upregulation). Related to Figure 3C and 4A.

**Table S2**. The phosphorylation profiling of broad-scope proteins in HL7702 transduced with PPM1A shRNA. Red indicated the elevated protein phosphorylation (>1.5-fold upregulation). Related to Figure 3C.

**Table S3.** The overlapped proteins phosphorylation (>1.5-fold upregulation) after PPM1B knockdown and PPM1A knockdown respectively. Related to Figure 3C.

**Table S4.** [Mass Spectrometry](https://www.sciencedirect.com/topics/biochemistry-genetics-and-molecular-biology/mass-spectrometry) of HA [Immunoprecipitation](https://www.sciencedirect.com/topics/medicine-and-dentistry/immunoprecipitation) in HL7702 cells overexpressed HA-PPM1B and blank vector respectively, Related to Figure 4B and 4C.

**Table S5.** The phosphorylation profiling of broad-scope proteins in HL7702 with HN252 treatment. Red indicated the elevated protein phosphorylation (>1.5-fold upregulation). Related to Figure 4A and 4B.

**Table S6.** GO and KEGG enrichment analysis of proteins differentially regulated by HN252 treatment (>1.5-fold increased phosphorylation after 2 hours exposure). Red indicated the enrichments overlapped with that of PPM1B knockdown. Related to Figure 4D and 4E.

**Table S7.** GO and KEGG enrichment analysis of proteins differentially regulated by PPM1B knockdown (>1.5-fold increased). Red indicated the enrichments overlapped with that of HN252 treatment. Related to Figure S6.

**Table S8.** Antibodies and commercial reagents used in this study.
